# Bioinspired Quinoline-2 Derivatives Based on the Natural Alkaloid 2-Phenylquinoline from *Conchocarpus longifolius* (A.St.-Hil.) Kallunki & Pirani: Pharmacological Evaluation of Their Gastroprotective Potential †

**DOI:** 10.3390/molecules31162815

**Published:** 2026-08-13

**Authors:** Sérgio Fallone de Andrade, Eduardo Breviglieri, Ivan Limachi, Luisa Mota da Silva, Thaise Boeing, Lincon Bordignon Somensi, Olov Sterner, Alberto Gimenez, Valdir Cechinel Filho

**Affiliations:** 1 Research Institute for Medicines (iMed.ULisboa), Faculty of Pharmacy, Universidade de Lisboa, 1649-003 Lisbon, Portugal; 2 Department of Pharmacy, Pharmacology and Health Technologies, Faculty of Pharmacy, Lisbon University, 1649-003 Lisbon, Portugal; 3 Programa de Pós-Graduação em Ciências Farmacêuticas, Núcleo de Investigações Químico-Farmacêuticas (NIQFAR), Universidade do Vale do Itajaí—UNIVALI, Itajaí 88302-901, SC, Brazil; breviglieri@univali.br (E.B.); boeing.thaise@univali.br (T.B.); cechinel@univali.br (V.C.F.); 4 Faculdade de Medicina Estácio de Jaraguá do Sul, Jaraguá do Sul 89265-100, SC, Brazil; 5 Center for Analysis and Synthesis (CAS), Lund University, 221 00 Lund, Sweden; ilimachi@umsa.bo (I.L.);; 6 Department of Pharmacology, Laboratory of Pharmacology Applied to the Gastrointestinal Tract and Its Interactions, Universidade Federal de Santa Catarina, Florianopolis 88040-900, SC, Brazil; luisa.mota@ufsc.br; 7 Graduate Program in Development and Society, Universidade Alto Vale do Rio do Peixe (UNIARP), Rua Victor Baptista Adami, 800, Caçador 89500-199, SC, Brazil; lincon.bordignon@uniarp.edu.br; 8 Instituto de Investigaciones Fármaco Bioquímicas (IIFB), Universidad Mayor de San Andrés, La Paz, Bolivia; ajgimenez@umsa.bo

**Keywords:** *Conchocarpus longifolius*, alkaloid, quinolines, gastroprotection, organic synthesis

## Abstract

The treatment of gastric ulcers based on antisecretory drugs is often associated with side effects and high recurrence rates, reinforcing the need for new therapeutic alternatives. Medicinal chemistry guided by natural prototypes represents a productive strategy in this context. The antiulcer potential of 2-phenylquinoline (2-PQ), an alkaloid from *Conchocarpus longifolius* (A.St.-Hil.) Kallunki & Pirani (syn. *Galipea longiflora* Krause), has been previously reported by our research group. In the present work, four 2-PQ derivatives were synthesized—2,4-diphenylquinoline (1), 2-(4-methoxyphenyl) quinoline (2), 2-phenylquinolin-4-ol (3), and 4-methoxy-2-phenylquinoline (4)—and evaluated for gastroprotective activity in the HCl/ethanol-induced gastric ulcer model in mice. The quinoline derivatives were prepared mainly by trifluoroacetic acid-catalyzed condensations of aminated benzaldehyde or benzophenone precursors with the corresponding ketones under reflux at 100 °C, affording yields of 68–94%. Compound 3 was synthesized via a two-step sequence involving the acylation of 2-aminobenzophenone followed by base-induced cyclization, providing an 85% yield. Compound 4 was obtained by O-methylation of compound 3 using iodomethane (MeI) and potassium carbonate (K_2_CO_3_) in dimethylformamide (DMF), affording a 95% yield. So, this study provides the first comparative analysis linking structural modifications to gastroprotective activity in synthetic quinoline derivatives inspired by 2-phenylquinoline, a natural alkaloid previously shown to exert gastroprotective effects. Carbenoxolone (200 mg/kg, p.o., positive control) inhibited gastric lesion formation by 94.1%. The synthetic quinoline derivatives also showed significant gastroprotective activity after oral administration (30 mg/kg), reducing ulcer area by 72.4% (compound **1**), 76.1% (compound **2**), 49.1% (compound **3**), and 66.1% (compound **4**). Compounds **2**, **3**, and **4** further retained efficacy following intraperitoneal administration (3 mg/kg), whereas compound **1** was inactivated by this route. Overall, compound **4** exhibited the greatest efficacy, significantly reducing the gastric lesion area, decreasing lipid hydroperoxide (LOOH) and tumor necrosis factor-alpha (TNF-α) levels, and increasing glutathione (GSH) content in ulcerated gastric tissue. These findings suggest that its gastroprotective effects are mediated by antioxidant and anti-inflammatory mechanisms, identifying compound **4** as the most promising candidate for the prevention and treatment of peptic ulcers. Its promising pharmacological profile warrants further investigation to elucidate its molecular mechanisms of action, assess its safety and efficacy in additional preclinical studies, and explore its potential for future therapeutic development.

## 1. Introduction

Peptic ulcer disease (PUD) is a chronic condition characterized by injury of the gastrointestinal mucosa, with gastric ulcer representing the most prevalent disorder of the upper digestive tract [1,2]. The disease affects approximately 10% of the global population [3,4], with recurrence rates as high as 60% in Western countries [5,6].

PUD results from an imbalance between aggressive luminal factors and mucosal defense mechanisms, involving a complex network of molecular pathways that regulate both tissue injury and repair. Mucosal damage is driven by excessive exposure to gastric acid and pepsin, *Helicobacter pylori* virulence factors, and NSAID-induced suppression of prostaglandin synthesis, which together activate inflammatory cascades mediated by TNF-α, interleukins, and prostaglandins [7]. These mediators increase oxidative stress through lipid peroxidation, elevating LOOH levels and depleting endogenous antioxidants such as GSH, thereby weakening epithelial integrity and microvascular homeostasis. Disruption of mucosal blood flow is further amplified by impaired signaling of nitric oxide (NO) and hydrogen sulfide (H_2_S), gaseous mediators essential for vasodilation, leukocyte inhibition, and maintenance of the gastric barrier [8]. Conversely, ulcer healing depends on the coordinated activation of growth factor-driven pathways—including epidermal growth factor (EGF), transforming growth factor-α (TGF-α), vascular endothelial growth factor (VEGF), hepatocyte growth factor (HGF), and basic fibroblast growth factor (bFGF)—which promote epithelial proliferation, migration, angiogenesis, and extracellular matrix remodeling [9].

Current pharmacological treatment of PUD relies primarily on histamine H_2_ receptor antagonists (H_2_RAs) and proton pump inhibitors (PPIs) such as omeprazole and pantoprazole, often combined with antibiotics for *H. pylori* eradication [6,10]. Despite their widespread use, several studies have indicated that H_2_RAs and PPIs present important adverse effects [11], including increased risk of dementia, gastric cancer, atrophic gastritis, and hypergastrinemia with long-term use [12,13]. Furthermore, high ulcer recurrence rates after treatment discontinuation reinforce the need for new therapeutic alternatives with improved efficacy and safety profiles [14].

In this context, medicinal plants have been used for treating gastric ulcers for millennia, and plant-derived compounds have emerged as promising antiulcer agents with fewer adverse effects than conventional drugs [1,15]. *Conchocarpus longifolius* (A.St.-Hil.) Kallunki & Pirani (syn. *Galipea longiflora Krause*), a Rutaceae from the Bolivian Amazon known as Evanta, is used in traditional folk medicine and has yielded 2-phenylquinoline (2-PQ), an alkaloid previously demonstrated by our research group to exert significant gastroprotective activity alongside anti-inflammatory, immunomodulatory, and antiparasitic effects [14,16,17].

Medicinal chemistry guided by natural prototypes represents an interesting strategy, allowing structural optimization of bioactive scaffolds to enhance potency and selectivity. In recent years, advances in the medicinal chemistry of quinolines have reinforced the potential of this heterocyclic nucleus as a platform for the development of antioxidant, anti-inflammatory and gastroprotective agents. Quinoline derivatives have been explored as modulators of targets involved in the inflammatory response, including TLR4/MD-2 inhibitors and regulators of kinases associated with tissue damage progression, further supporting the therapeutic appeal of this scaffold for gastrointestinal conditions [18,19,20,21]. In the context of optimizing natural prototypes, structural modifications of quinoline alkaloids—including the introduction of hydroxyl, methoxyl, and aryl groups—have been employed to improve biological activity and safety profiles, aligning with modern drug discovery approaches based on natural products [1,20,22].

Considering the previously demonstrated gastroprotective effects of 2-PQ and the well-established influence of structural modifications on the biological activity of quino-line scaffolds, this study was designed to determine how defined changes in the 2-phenylquinoline scaffold influence its gastroprotective efficacy. To achieve this, we synthesized four derivatives structurally related to 2-PQ—2,4-diphenylquinoline (1), 2-(4-methoxyphenyl)quinoline (2), 2-phenylquinolin-4-ol (3), and 4-methoxy-2-phenylquinoline (4)—and evaluated their ability to prevent HCl/ethanol-induced gastric lesions in mice. A further objective was to gain mechanistic insights into their biological effects by quantifying key oxidative and inflammatory markers in ulcerated gastric tissue, such as lipid hydroperoxides (LOOHs), reduced glutathione (GSH), and tumor necrosis factor-α (TNF-α) levels.

## 2. Results

### 2.1. Synthesis of 2-PQ and Quinoline Derivatives

The successful synthesis of 2-phenylquinoline (2-PQ) by our research group represents an important step toward its sustainable production and further pharmacological investigation, as it ensures independence from plant extraction and avoids the environmental impact associated with bark harvesting, which may negatively affect the conservation of *Conchocarpus longifolius*. In this context, 2-PQ was obtained in high yield (87%) through the condensation of 2-aminobenzaldehyde (1 mmol) with acetophenone (1 mmol) in the presence of trifluoroacetic acid (TFA, 1.5 mmol), as illustrated in Scheme S1 (available in the Supplementary Material). The synthesis of the four quinoline derivatives was previously described by [17] and afforded yields of 94%, 68.3%, 85%, and 95% for compounds **1**, **2**, **3**, and **4**, respectively, as described in Scheme S2 (available in the Supplementary Material). Compound **4**, although synthesized in the present study, has been reported as a naturally occurring alkaloid and is less abundant than 2-PQ in *Conchocarpus longifolius*.

### 2.2. Chemical Characterization

The chemical characterization was done by NMR, and purity of all compounds was 95% higher.

#### NMR-Data

2,4-diphenyl-quinoline (**1**)

White crystal, mp: 112–120 °C. 1H NMR (400 MHz, CDCl3): δ 7.63–7.46 (9H, m), 7.77 (1H, ddd, *J* = 8.3, 6.9, 1.3 Hz), 7.87 (1H, s), 7.85 (1H, dd, *J* = 8.4, 0.9 Hz), 8.26–8.20 (2H, m), 8.32 (1H, d, *J* = 7.8 Hz). 13C NMR (100.6 MHz, CDCl3): δ 119.42, 125.67, 125.79, 126.37, 127.62, 128.44, 128.63, 128.87, 129.38, 129.57, 130.13, 138.42, 139.6, 143.2, 143.99, 148.9, 149.25, 156.92. MS: *m*/*z*: (281)

2-(4-methoxy-phenyl)-quinoline (**2**)

White crystal, mp: 125–126 °C. 1H NMR (400 MHz, CDCl3): δ 8.21 (1H, d, *J* = 8.7 Hz), 8.19–8.15 (3H, m), 7.86 (1H, d, *J* = 8.6 Hz), 7.83 (1H, dd, *J* = 8.1, 1.2 Hz), 7.74 (1H, ddd, 8.4, 6.9, 1.5 Hz), 7.53 (1H, ddd, 8.1, 6.9, 1.1 Hz), 7.08 (2H, m), 3.92 (OCH3, s). 13C NMR (100.6 MHz, CDCl3): δ 160.85, 156.91, 148.23, 136.72, 132.19, 129.63, 129.48, 128.93, 127.45, 126.92, 125.96, 118.59, 114.25, 55.42. MS: *m*/*z*: (235)

2-phenylquinoline-4-ol—4-hidroxy-2-phenyl-quinoline (**3**)

Solid yellow pale, mp: 245–247 °C. 1H NMR (400 MHz, DMSO-d6): δ 8.11 (1H, dd, *J* = 8.1, 1.2 Hz)7.91–7.84 (2H, m), 7.83 (1H, d, *J* = 8.6 Hz), 7,68 (1H, ddd, *J* = 8.4 Hz), 7.62–7.56 (3H, m), 7.35 (1H, t, *J* = 7.3 Hz), 6.34 (1H, S). 13C NMR (100.6 MHz, DMSO-d6): δ 107.77, 119.26, 123.74, 125.16, 127.93, 129.46, 130.92, 132.25, 141.03, 150.48, and 177.46. MS: *m*/*z*: (221)

4-metoxy-2-phenyl-quinoline (**4**)

Solid yellow pale, mp: 55–58 °C. 1H NMR (400 MHz, CDCl3): δ 8.22 (1H, dd, *J* = 8.3, 1.0 Hz), 8.17–8.11 (3H, m), 7.74 (1H, ddd, *J* = 8.4, 6.9, 1.5 Hz), 7.56 (2H, dd, *J* = 7.6, 1.3 Hz), 7.53–7.46 (2H, m), 7.2 (1H, s), 4.14 (3H, s). 13C NMR (100.6 MHz, CDCl3): δ 162.86, 158.88, 149.16, 140.39, 130.01, 129.09, 129.20, 128.8, 127.61, 125.43, 121.66, 120.40, 98.03, 55.69. MS: *m*/*z*: (235)

### 2.3. Gastroprotective Effects

**Figure 1:**
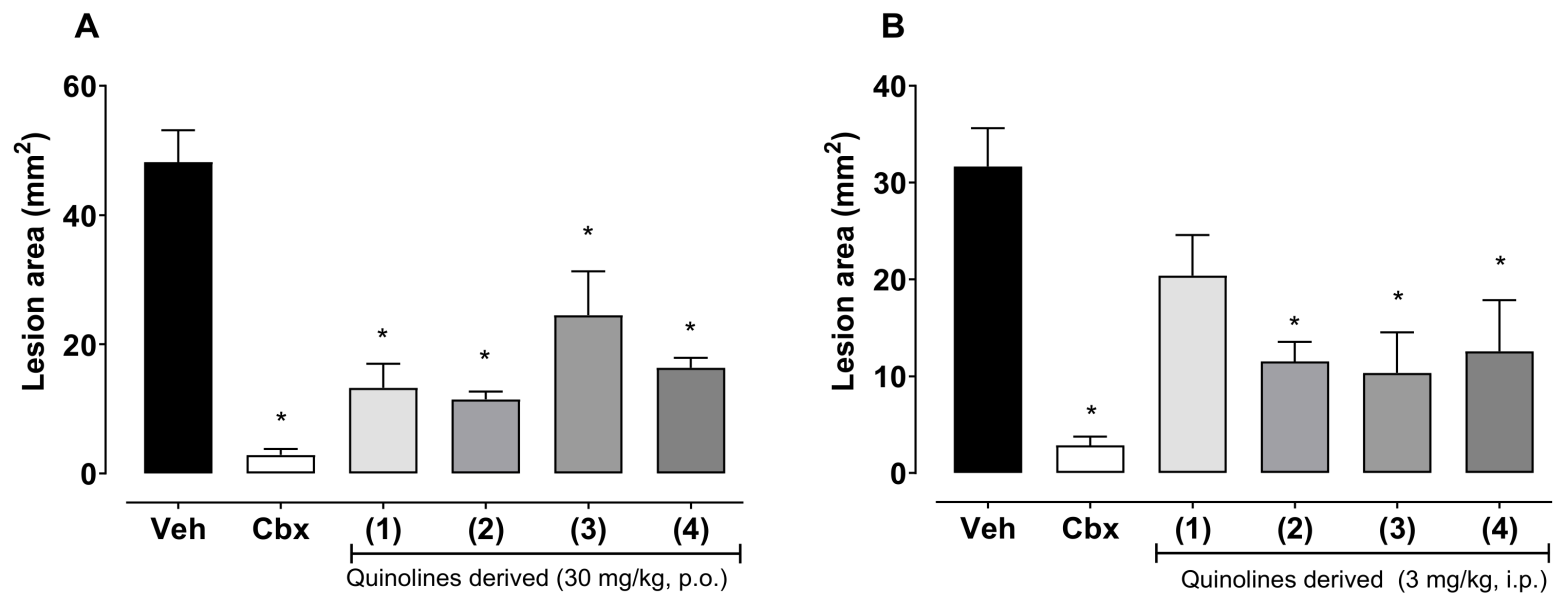
Gastroprotective effects of quinolines derived from 2-PQ on gastric ulcers induced by ethanol/HCl. (**A**) The animals were treated orally (v.o.) with vehicle (Veh: 10 mL/kg), carbenoxolone (Cbx, 200 mg/kg) or quinolines structurally related to 2-PQ: 2,4-diphenylquinoline (1), 2-(4-methoxyphenyl)quinoline (2), 2-phenylquinoline-4-ol (3) and 4-methoxy-2-phenylquinoline (4) (30 mg/kg); (**B**) animals were treated with the quinolines intraperitoneally (i.p) (3mg/kg). The results are expressed as mean ± SEM (n = 6), one way-ANOVA followed by Dunnett’s test. (* *p* < 0.05 compared to the Veh).

**Figure 2:**
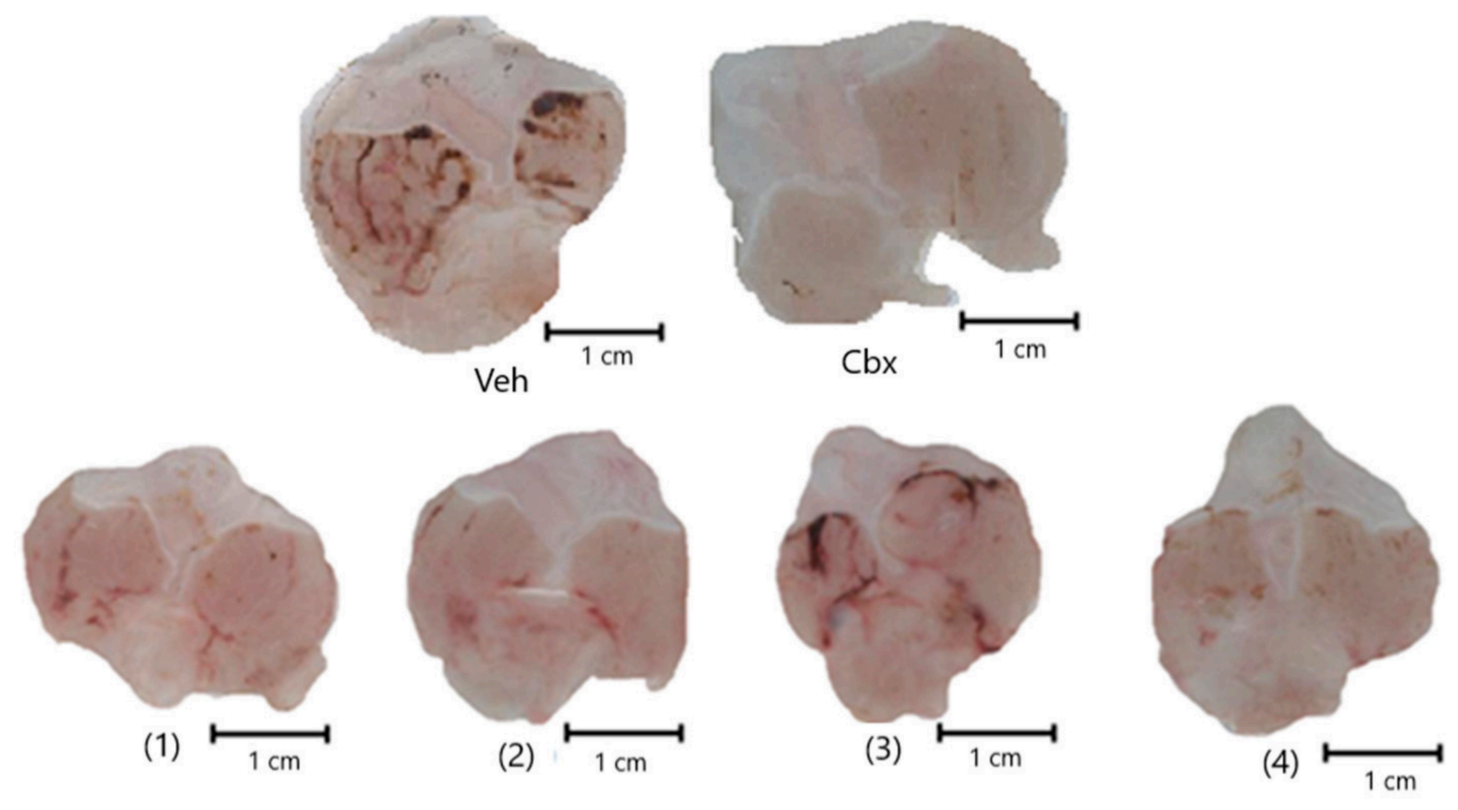
Representative images of the gastroprotective effect of 2-PQ decrease acute gastric lesions induced by ethanol/HCl in mice. Vehicle (Veh, 10 mL/kg), carbenoxolone (Cbx, 200 mg/kg—Positive control) or quinolines structurally related to 2-PQ: 2,4-diphenylquinoline (1), 2-(4-methoxyphenyl) quinoline (2), 2-phenylquinoline-4-ol (3) and 4-methoxy-2-phenylquinoline (4) (30 mg/kg).

The oral administration of acidified ethanol promoted gastric lesions in an extension of 48.2 ± 4.9 mm^2^ in the vehicle group. Carbenoxolone (200 mg/kg, p.o., positive control) inhibited the formation of gastric lesions by 94.1%. The oral administration of compounds **1**, **2**, **3**, and **4** at a dose of 30 mg/kg significantly reduced the area of gastric injury by 72.4%, 76.1%, 49.1%, and 66.1%, respectively, compared to the vehicle group. The gastroprotective effects of compounds **2**, **3**, and **4** were further confirmed following intraperitoneal administration at 3 mg/kg, whereas compound **1** was ineffective via this route. Representative images of the stomachs are presented in Figure 1 and Figure 2.

All animals were monitored throughout the experiment, and no signs of overt toxicity (e.g., changes in locomotor activity, grooming, posture, respiratory pattern, or mortality) were observed at the tested doses and routes of administration. The selection of the doses (30 mg/kg, p.o., and 3 mg/kg, i.p.) was based on previously reported studies using structurally related quinoline-based compounds that demonstrated biological activity in vivo without evident toxicity at comparable dose ranges [14,23].

### 2.4. Effects on Lipid Hydroperoxide (LOOH) Levels

**Figure 3:**
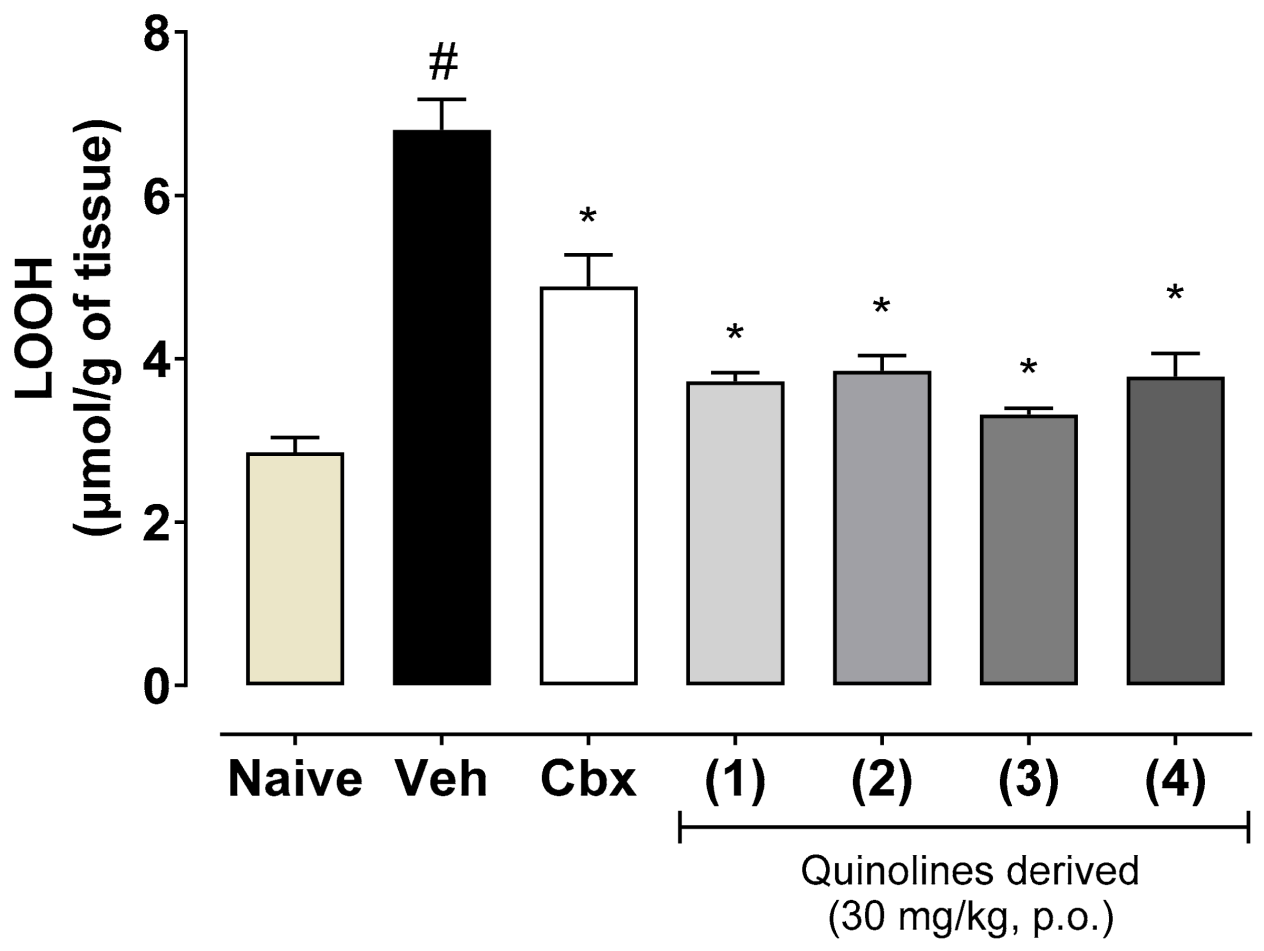
Effects of quinolines derived from 2-PQ on lipid hydroperoxides (LOOH) levels on acute gastric lesions induced by ethanol/HCl in mice. The animals were treated orally with vehicle (Veh: 10 mL/kg), carbenoxolone (Cbx, 200 mg/kg) quinolines structurally related to 2-PQ: 2,4-diphenylquinoline (1), 2-(4-methoxyphenyl) quinoline (2), 2-phenylquinoline-4-ol (3) and 4-methoxy-2-phenylquinoline (4) (30 mg/kg orally—p.o or 3 mg/kg intraperitoneally—i.p.). The results are expressed as mean ± SEM (n = 6), one way-ANOVA followed by Dunnett’s test. (* *p* < 0.05 compared to the Veh; # *p* < 0.05 compared to the naïve).

Biochemical assays performed in gastric tissue ulcerated by ethanol/HCl intake demonstrated that pretreatment with all tested quinoline derivatives significantly attenuated the increase in lipid hydroperoxide levels induced by the ulcerogenic agent, indicating a protective effect against ethanol-induced oxidative damage at the mucosal level (Figure 3).

### 2.5. Effects on Reduced Glutathione (GSH) Levels

**Figure 4:**
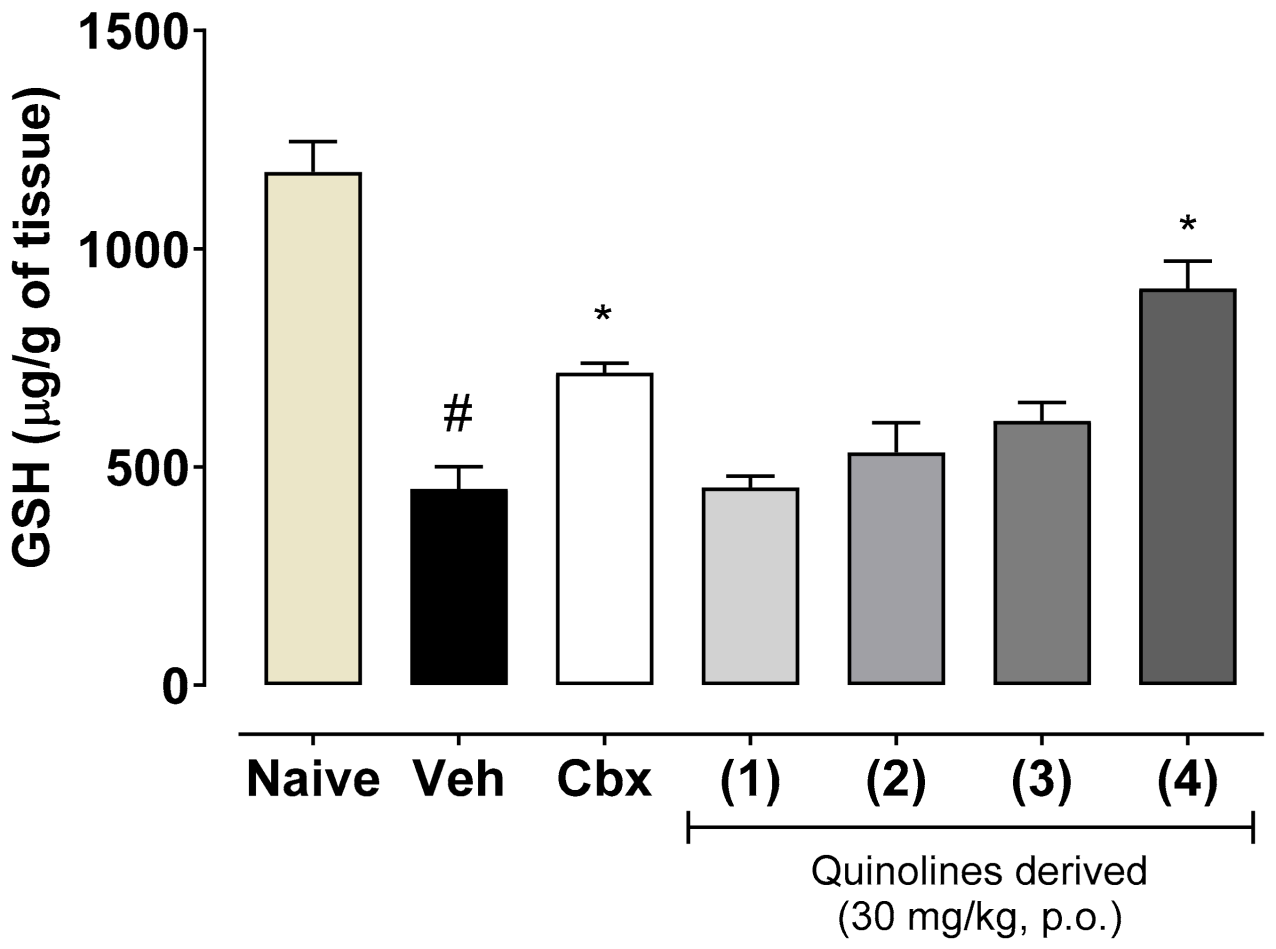
Effects of quinolines derived from 2-PQ on reduced gluthatione (GSH) levels. The animals were treated orally with vehicle (Veh: 10 mL/kg), carbenoxolone (Cbx, 200 mg/kg) quinolines structurally related to 2-PQ: 2,4-diphenylquinoline (1), 2-(4-methoxyphenyl) quinoline (2), 2-phenylquinoline-4-ol (3) and 4-methoxy-2-phenylquinoline (4) (30 mg/kg orally—p.o or 3 mg/kg intraperitoneally—i.p.). The results are expressed as mean ± SEM (n = 6), one way-ANOVA followed by Dunnett’s test. (* *p* < 0.05 compared to the Veh; # *p* < 0.05 compared to the naïve).

As shown in Figure 4, exposure to acidified ethanol markedly reduced GSH levels in the vehicle group (449.7 ± 51.23 µg/g of tissue) compared to naïve animals (1176.0 ± 69.17 µg/g of tissue). Pretreatment with carbenoxolone significantly restored GSH levels (716.3 ± 21.74 µg/g of tissue; * *p* < 0.05 vs. vehicle group). Compounds **1**, **2**, and **3** did not significantly restore GSH levels (453.3 ± 25.68, 533.4 ± 68.33, and 605.8 ± 41.57 µg/g of tissue, respectively). In contrast, compound **4** was the only derivative to produce a statistically significant increase in GSH content (908.7 ± 63.08 µg/g of tissue; * *p* < 0.05 vs. vehicle group), approaching values observed in naïve animals.

### 2.6. Effects on TNF-α Levels

**Figure 5:**
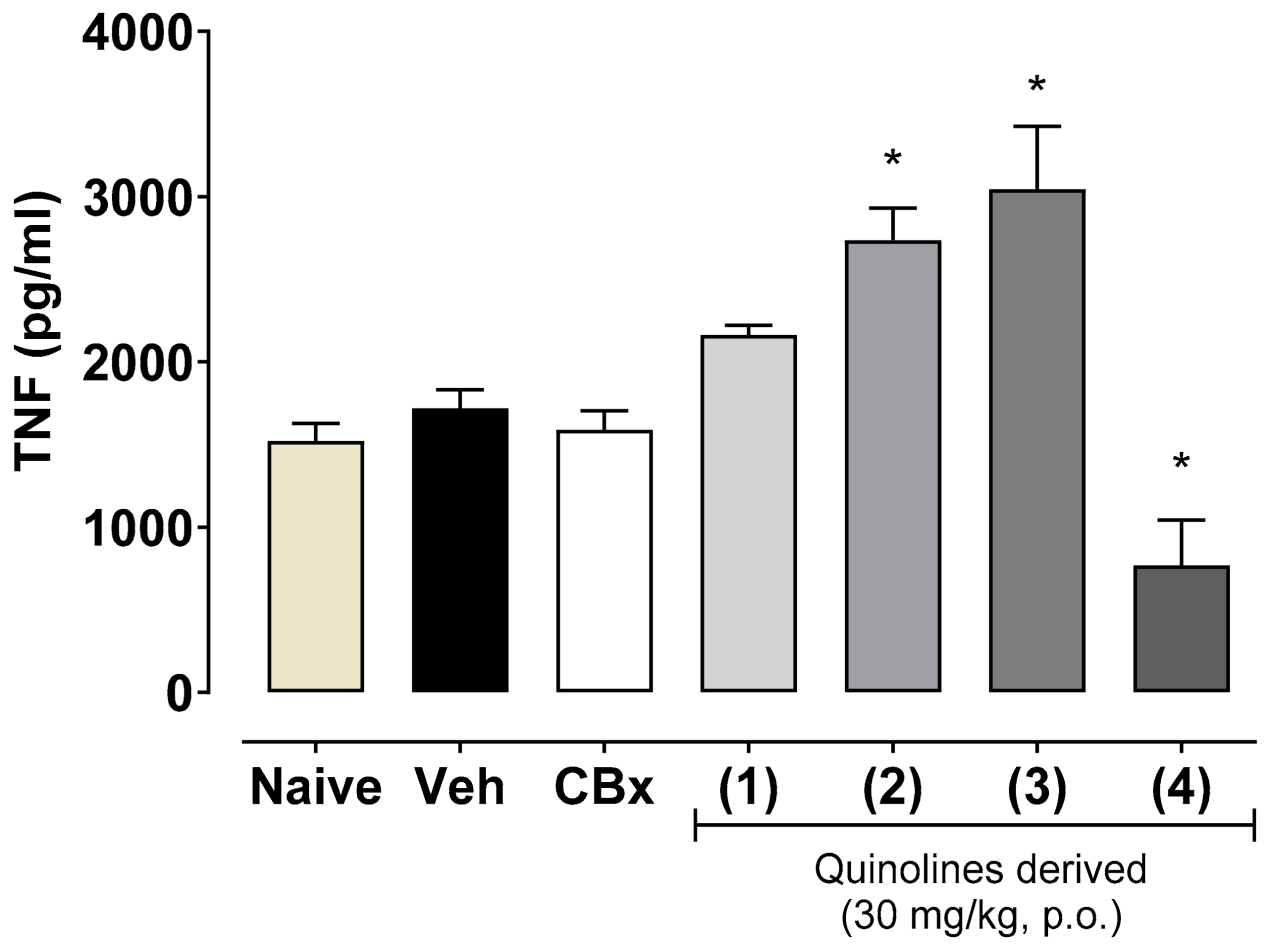
Effects of 2-PQ and the derivatives on TNF-α levels. The animals were treated orally with vehicle (Veh: 10 mL/kg), carbenoxolone (CBx, 200 mg/kg) quinolines structurally related to 2-PQ: 2,4-diphenylquinoline (1), 2-(4-methoxyphenyl) quinoline (2), 2-phenylquinoline-4-ol (3) and 4-methoxy-2-phenylquinoline (4) (30 mg/kg orally—p.o or 3 mg/kg intraperitoneally—i.p.). The results are expressed as mean ±SEM (n = 6), one way-ANOVA followed by Dunnett’s test. (* *p* < 0.05 compared to the Veh).

TNF-α levels in the gastric mucosa of animals in the vehicle group were 1719.0 ± 111.8 pg/mL. Compounds **1**, **2**, and **3**, despite exhibiting significant macroscopic gastroprotective effects via oral administration, failed to significantly reduce TNF-α levels in ulcerated gastric tissue. In contrast, compound **4** decreased TNF-α levels by 55.3% (* *p* < 0.05 vs. vehicle group), as shown in Figure 5.

## 3. Discussion

The quinoline derivatives evaluated in this study exhibited marked gastroprotective activity in the HCl/ethanol-induced ulcer model, suggesting that the quinoline scaffold accommodate structural modifications while retaining biological relevance. These findings are consistent with prior reports highlighting quinoline as a versatile scaffold with broad pharmacological potential [24,25,26] as well as with the previously described gastroprotective activity of the natural alkaloid 2-phenylquinoline (2-PQ) [14]. Verbanac and collaborators [27] (Verbanac 2016) evaluated the antioxidant, cytotoxic, and bactericidal activity of new 2-PQ analogs with derivatization by aromatic substituents at position 4 and indicated that specific alterations are associated with the increase or decrease in the measured effects, further supporting the importance of structural features at this position.

Ethanol-induced gastric injury follows a well-characterized pathogenic cascade beginning with disruption of the mucosal barrier, solubilization of mucus, and increased epithelial permeability [28,29]. This is followed by excessive generation of reactive oxygen species (ROS), lipid peroxidation, depletion of endogenous antioxidants such as GSH, and amplification of proinflammatory mediators including TNF-α [30,31,32,33,34]. Many gastroprotective agents exert their effects by attenuating oxidative stress, restoring antioxidant defenses, and modulating inflammatory signaling. Integrating our biochemical and macroscopic findings within this mechanistic framework reveals distinct pharmacological profiles among the tested derivatives.

The gastroprotective effects of all four compounds after oral administration (30 mg/kg) were evident. However, compound **1** did not show activity when administered intraperitoneally (3 mg/kg, Figure 1B). The inclusion of both oral and intraperitoneal routes aimed to generate complementary information on compound efficacy under distinct experimental conditions and to assess how structural modifications of the 2-phenylquinoline (2-PQ) scaffold influence gastroprotective activity. This design allowed evaluation of whether different substituents preserve or alter the pharmacological profile of the parent compound depending on the administration route, although it does not support definitive conclusions regarding absorption, bioavailability, or systemic disposition. A lower dose was selected for intraperitoneal administration because this route bypasses factors that influence drug exposure after oral dosing, such as gastrointestinal absorption and first-pass metabolism. Thus, the doses were optimized to detect pharmacological activity within each route rather than to enable direct quantitative comparisons between them.

Compounds **1**, **3**, and **4** share structural modifications at carbon-4 of the quinoline ring, and compound **1** contains the largest substituent at this position—a phenyl group. This bulky substituent may influence the physicochemical and pharmacological properties of the molecule, which could potentially contribute to reduced systemic bioavailability. Therefore, the activity of compound **1** after oral administration may reflect a localized interaction with the gastric mucosa, a phenomenon frequently observed for orally administered gastroprotective agents, rather than a mechanism dependent on systemic exposure.

All four compounds reduced LOOH levels, suggesting that the quinoline scaffold may be associated with antioxidant effects capable of limiting lipid peroxidation regardless of structural modification. Lipid hydroperoxides are established biomarkers of lipid peroxidation and oxidative tissue injury [35,36], including oxidative damage in the gastric mucosa [32,37]. The observed reduction suggests that the quinoline derivatives evaluated in this study are associated with reduced oxidative damage following ethanol exposure. However, the effects of quinoline derivatives on mucosal GSH levels and TNF-α levels have been variable.

TNF-α plays a central role in the initiation and amplification of acute inflammatory responses in the gastric mucosa, including neutrophil recruitment and propagation of tissue injury [38]. Elevated TNF-α levels are associated with the inflammatory phase of gastric ulceration and may contribute to the initiation and amplification of mucosal injury [39], whereas lower TNF-α levels are associated with improved mucosal healing. GSH is a key endogenous antioxidant in the gastric mucosa, where it helps maintain redox homeostasis, detoxify reactive oxygen species, and support mucosal cytoprotection; accordingly, reduced mucosal GSH levels have been associated with gastric ulceration and delayed healing [40,41,42].

The structural basis for these differences is consistent with SAR trends observed in quinoline pharmacology, where steric bulk at key positions can modulate bioavailability and weaken receptor or enzyme interactions [27,43,44]. Compounds **1**, **3**, and **4** share modifications at carbon 4 of the quinoline ring, but compound **1** contains the bulkiest substituent—a phenyl group. Such bulky substituents at pharmacophoric positions may influence physicochemical properties and exposure profiles [45,46], potentially affecting the ability of compounds to modulate cellular responses associated with oxidative stress and inflammation. Consequently, compound **1** was effective only after oral administration, suggesting that its gastroprotective activity may involve local interactions with the gastric mucosa rather than effects requiring measurable systemic exposure. In contrast, compound **4** carries the smallest substituent at this position—a methoxy group—which may preserve a more favorable steric environment for interaction with biological targets involved in mucosal protection. In addition to steric considerations, the methoxy group is an electron-donating substituent that may modify the electronic distribution of the quinoline scaffold, potentially influencing its interaction with molecular targets associated with oxidative stress and inflammatory pathways. Methoxy substitution has also been associated with favorable changes in lipophilicity and membrane permeability in aromatic pharmacophores, which may contribute to improved pharmacological performance through enhanced tissue exposure [47,48,49]. Although these hypotheses were not directly investigated in the present study, they provide plausible explanations for the superior activity observed for compound **4**. This structural feature may contribute to the observed ability of compound **4** to increase GSH levels and reduce TNF-α levels, which may be associated with its gastroprotective activity following both oral and intraperitoneal administration.

Compound **2**, which carries a substituent on the phenyl ring at position 2 of the quinoline nucleus rather than at carbon 4, preserved macroscopic gastroprotection but did not significantly modify biochemical markers associated with oxidative stress and inflammation. This dissociation suggests that modifications outside the C4 region may maintain macroscopic protection while producing different profiles of biochemical responses related to oxidative stress and inflammation. Similarly, compounds **1** and **3** produced macroscopic protection without significant changes in GSH or TNF-α levels, suggesting that their effects may depend on mechanisms independent of antioxidant replenishment or inflammatory suppression.

The inability of compounds **1**, **2**, and **3** to significantly reduce TNF-α—despite their macroscopic gastroprotective effects—suggests that their gastroprotective effects may occur independently of significant modulation of TNF-α-associated inflammatory pathways. In contrast, compound **4** decreased TNF-α levels by 55.3%, suggesting that modulation of inflammatory responses may be associated with its gastroprotective profile in the ulcerated gastric mucosa. This dissociation between macroscopic gastroprotection and inflammatory modulation underscores the importance of complementary biochemical analyses to accurately characterize gastroprotective mechanisms and highlights the limitations of relying solely on lesion area as an outcome measure.

Although the present findings provide initial insights into the influence of structural modifications on the gastroprotective activity of the 2-PQ scaffold, the structure–activity relationship remains preliminary due to the limited number of derivatives evaluated and the absence of dose–response analyses. Future studies including additional analogues, the parent 2-PQ compound, and broader pharmacological characterization will be required to establish more comprehensive SAR relationships. Beyond the limitations of the structure–activity analysis, the present study is also limited to an acute HCl/ethanol-induced gastric ulcer model, which reproduces key features of acute mucosal injury but does not fully reflect the complexity of chronic gastric ulcer disease. Therefore, although the results suggest promising gastroprotective activity, additional studies using chronic ulcer models, together with comprehensive toxicological and pharmacokinetic evaluations, will be essential to further establish the therapeutic potential and translational relevance of these compounds.

Taken together, these findings support the hypothesis that quinoline derivatives may exert gastroprotective effects in association with reduced oxidative damage, increased mucosal antioxidant capacity, and modulation of TNF-α levels, although additional studies are required to establish their precise mechanistic contribution. Compound **4**, bearing the smallest substituent at this position, was the only derivative associated with simultaneous reductions in lipid peroxidation, increased GSH levels, and decreased TNF-α levels, displaying a more complete biochemical and gastroprotective profile. These findings highlight the contribution of C4 substitution in determining both the extent and diversity of biological activity within this class of compounds.

## 4. Materials and Methods

### 4.1. Plant Material and Extraction

The procedures for extracting and isolating the alkaloid fraction were performed from *G. longiflora* (Gl) bark, chromatographed on a silica gel column with an ethyl acetate gradient in n-hexane. From 1 g of alkaloid fraction, 55 mg of 2-PQ was obtained and identified by thin-layer chromatography (co-TLC) and nuclear magnetic resonance (NMR) spectral data compared to an authentic sample [14]. The collection of the plant and the extraction and isolation procedures were carried out in the Bolivian province of Nor Yungas; the material was taxonomically identified by comparison with voucher samples (AS49 and SD17) deposited at the National Herbarium, PNL, La Paz, Bolivia. All chemicals and reagents used in synthesis and biological assays have been purchased from (Merck, Darmstadt, Germany).

### 4.2. Synthesis

**Figure 6:**
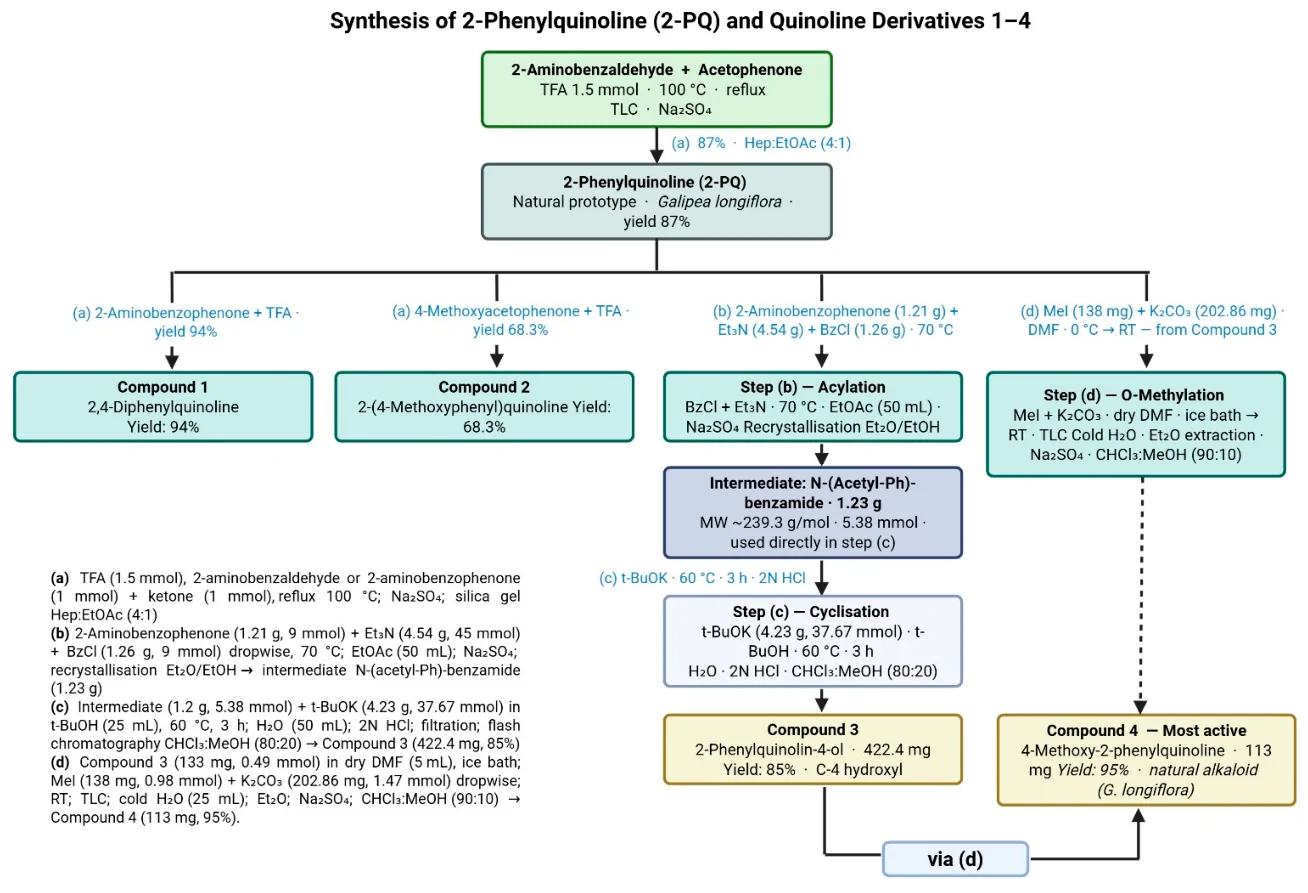
Synthetic pathway for 2-Phenylquinoline (2-PQ) and derivatives 1–4. The scheme illustrates the sequential reactions leading to quinoline derivatives with distinct substituents and yields: (a) condensation of 2-aminobenzaldehyde or 2-aminobenzophenone with ketones under TFA reflux to form 2-phenylquinoline and analogues; (b) acylation of 2-aminobenzophenone to yield N-(acetyl-Ph)-benzamide; (c) cyclisation with t-BuOK/t-BuOH affording 2-phenylquinolin-4-ol; and (d) O-methylation with MeI/K_2_CO_3_ producing 4-methoxy-2-phenylquinoline, the most active derivative. Reaction conditions and yields are indicated for each step.

The synthesis of 2-phenylquinoline (2-PQ) was accomplished via acid-catalyzed condensation as described in Figure 6. Briefly, 2-aminobenzaldehyde (1 mmol) and acetophenone (1 mmol) were combined in the presence of trifluoroacetic acid (TFA, 1.5 mmol) and stirred under reflux at 100 °C until complete consumption of starting materials, as monitored by thin-layer chromatography (TLC) (Merck, Darmstadt, Germany). Upon completion, the reaction mixture was allowed to cool to room temperature, and the organic phase was dried over anhydrous Na_2_SO_4_, filtered, and concentrated under reduced pressure. The crude product was purified by silica gel column chromatography using heptane and ethyl acetate (4:1, *v*/*v*) as the mobile phase to afford 2-PQ in 87% yield. The four quinoline derivatives structurally related to 2-PQ were subsequently synthesized following the procedures previously reported by [17], with minor modifications. Compounds **1** and **2** were obtained by condensation of the appropriate 2-aminobenzaldehyde or 2-aminobenzophenone derivative (1 mmol) with the corresponding ketone (1 mmol) in the presence of TFA (1.5 mmol), under reflux at 100 °C, with reaction progress monitored by TLC. Upon completion, the organic phase was dried over anhydrous Na_2_SO_4_, filtered, evaporated under reduced pressure, and the residue purified by silica gel column chromatography (heptane:ethyl acetate, 4:1, *v*/*v*), affording compounds **1** and **2** in yields of 94% and 68.3%, respectively. Compound **3** was prepared in two sequential steps. First, a solution of 2-aminobenzophenone (1.21 g, 9 mmol) and triethylamine (Et_3_N, 4.54 g, 45 mmol) was treated with benzoyl chloride (1.26 g, 9 mmol), added dropwise at 70 °C and maintained at this temperature until complete reaction, as confirmed by TLC. The mixture was poured into water, extracted with ethyl acetate (50 mL), and the organic phase was dried over anhydrous Na_2_SO_4_ and concentrated under reduced pressure. The resulting solid was recrystallized from diethyl ether/ethanol to yield 1.23 g of the intermediate N-(acetyl-phenyl)-benzamide. In the subsequent step, this intermediate (1.2 g, 5.38 mmol) was dissolved in tert-butanol (25 mL), and potassium tert-butoxide (t-BuOK, 4.23 g, 37.67 mmol) was added at 60 °C. The reaction was maintained for 3 h, after which the mixture was poured into water (50 mL) and acidified with 2 N HCl to precipitate the product. The solid was collected by filtration and purified by flash chromatography (chloroform:methanol, 80:20, *v*/*v*) to afford 422.4 mg of compound **3** in 85% yield. Finally, compound **4** was obtained by O-methylation of compound **3** (133 mg, 0.49 mmol), dissolved in dry dimethylformamide (DMF, 5 mL) under an ice bath, to which iodomethane (MeI, 138 mg, 0.98 mmol) and anhydrous potassium carbonate (K_2_CO_3_, 202.86 mg, 1.47 mmol) were added dropwise. The reaction mixture was allowed to warm to room temperature and stirred until complete conversion, monitored by TLC. Cold water (25 mL) was then added, the product extracted with diethyl ether, and the organic phase dried over anhydrous Na_2_SO_4_, filtered, and concentrated under reduced pressure. The crude product was purified by silica gel column chromatography (chloroform:methanol, 90:10, *v*/*v*) to afford 113 mg of pure compound **4** in 95% yield.

### 4.3. Chemical Characterization

All chemicals were obtained from commercial suppliers of analytical grade. Mass spectrometry (MS) electrospray ionization (ESI) spectra were recorded with a micromass quadrupole-time of flight (QTOF) micro spectrometer. 1H-NMR spectra were assigned using 1D- and 2D-methods with a Varian Gemini (Palo Alto, Santa Clara, CA, USA) at 300 MHz (1H) and at 75 MHz (13C). Chemical shifts for ^1^H and ^13^C are given in ppm with the signal for residual solvent (CHCl3) as reference. The reactions were monitored with TLC using alumina plates coated with silica gel (Merck, Darmstadt, Germany) and visualized using either UV light or by charring with vanillin. Chromatography column was performed with 60 Å 30–75 µm Silica gel.

### 4.4. Animals

Male Swiss mice (*Mus musculus*), weighing 25–35 g, were obtained from the institutional animal facility and maintained under controlled environmental conditions (temperature 22 ± 2 °C, relative humidity 50–60%, 12 h light/dark cycle) with free access to standard rodent chow and water ad libitum. Prior to experimental procedures, animals were acclimatized for at least 7 days. For the induction of gastric lesions, mice were fasted for 12 h with free access to water. All procedures followed the ethical principles of animal experimentation established by CONCEA (National Council for the Control of Animal Experimentation) and were approved by the Ethics Committee on Animal Use of the Universidade do Vale do Itajaí, Santa Catarina, Brazil (approval number CEUA: 38/14p), conducted in accordance with the Principles of Laboratory Animal Care.

### 4.5. Ethanol-Induced Gastric Ulcer Model

The gastroprotective activity of 2-PQ-derived compounds was assessed using the acidified ethanol-induced gastric ulcer model, with minor modifications from the method originally described by [50]. A priori sample size was determined based on previous studies using this model, assuming a 30–40% reduction in lesion area as biologically relevant, a standard deviation of 20%, α = 0.05, and power (1 − β) = 0.8, resulting in a minimum of six animals per group. Animals were randomly allocated to experimental groups ensuring equal group sizes (n = 6). Allocation concealment was maintained by an independent researcher not involved in data acquisition. All treatments were prepared and coded by a separate investigator to ensure blinding. Investigators responsible for ulcer induction, tissue processing, and outcome assessment were blinded to group allocation throughout the experiment and analysis. Treatments administered by gavage (p.o.) included the vehicle group, which received an aqueous solution of 1% Tween 80 (10 mL/kg), the positive control group, which received carbenoxolone (200 mg/kg), and groups treated with synthetic quinoline derivatives 1, 2, 3, and 4 at a dose of 30 mg/kg. For intraperitoneal administration (i.p.), the same groups have been used and synthetic quinoline derivatives (1–4) were administered at a dose of 3 mg/kg. One hour after pretreatment, gastric lesions were induced by oral administration of an ulcerogenic solution consisting of 60% ethanol acidified with 0.03 M HCl (10 mL/kg, p.o.). Animals were euthanized by CO_2_/O_2_ inhalation one hour after ulcer induction. The stomachs were immediately excised, rinsed with saline, and opened along the greater curvature. The total hemorrhagic lesion area (mm^2^) was quantified using EARP^®^ image analysis software version 1. Gastric tissue samples were snap-frozen and stored at −80 °C for biochemical analyses.

### 4.6. Biochemical Analysis

#### 4.6.1. Determination of Lipid Hydroperoxide (LOOH) Levels

Gastric tissue samples were homogenized in ice-cold 200 mM phosphate buffer (pH 6.5) and the resulting homogenates were immediately used for quantification of lipid hydroperoxide levels using the ferrous oxidation–xylenol orange (FOX) assay, as previously described [51]. Results were expressed as µg of GSH per gram of tissue (µmol/g of tissue) and presented as mean ± SEM (n = 6).

#### 4.6.2. Determination of Reduced Glutathione (GSH) Levels

Gastric mucosal samples were processed immediately after collection and kept on ice throughout all procedures to prevent oxidative degradation of GSH. Tissue samples were homogenized in ice-cold 200 mM potassium phosphate buffer (pH 6.5) at a ratio of 100 mg tissue per mL of buffer. The resulting homogenates were centrifuged at 10,000× *g* for 10 min at 4 °C, and supernatants were collected for analysis. GSH content was determined spectrophotometrically using Ellman’s reagent (5,5′-dithiobis-2-nitrobenzoic acid; DTNB), according to the method described by [52]. Results were expressed as µg of GSH per gram of tissue (µg/g of tissue) and presented as mean ± SEM (n = 6).

#### 4.6.3. Determination of Tumor Necrosis Factor alpha (TNF-α) Levels

TNF-α levels were quantified in ulcerated gastric tissue by sandwich enzyme-linked immunosorbent assay (ELISA), using a commercially available mouse cytokine ELISA kit (BD Biosciences, Franklin Lakes, NJ, USA), following the manufacturer’s instructions. Tissue samples were homogenized in ice-cold 200 mM potassium phosphate buffer (pH 6.5) and centrifuged at 10,000× *g* for 10 min at 4 °C. All samples were prepared using standardized amounts of gastric tissue and identical homogenization volumes, ensuring comparability among experimental groups. TNF-α concentrations were expressed as picograms per milliliter (pg/mL) and presented as mean ± SEM (n = 6).

### 4.7. Statistical Analysis

All results are expressed as mean ± standard error of the mean (SEM). Data normality was assessed using the Shapiro–Wilk test prior to applying one-way ANOVA. Statistical comparisons were performed using one-way analysis of variance (ANOVA) followed by Dunnett’s post hoc test. Values of *p* < 0.05 were considered statistically significant relative to the ulcerated vehicle group. In the figures, * indicates *p* < 0.05 compared to the ulcerated vehicle group (Veh), whereas # indicates *p* < 0.05 compared to the naïve group. Analyses were performed using GraphPad Prism^®^ software version 10.5.0.

## 5. Conclusions

The four synthetic quinoline derivatives (1–4) exhibited gastroprotective activity in the HCl/ethanol-induced ulcer model in mice following oral administration, suggesting that the quinoline scaffold accommodates structural modification while maintaining biological activity. The results suggest that substituents at carbon-4 of the quinoline ring influence the magnitude of the gastroprotective response: bulkier groups, as in compound **1**, were associated with reduced systemic efficacy, whereas the smaller methoxy substituent in compound **4** corresponded to more consistent improvements across the evaluated parameters. Although all derivatives reduced macroscopic lesion area, only compound **4** produced concurrent changes in key biochemical markers, including lipid hydroperoxides, reduced glutathione, and TNF-α. In contrast, modifications at position 2, as in compound **2**, preserved macroscopic protection but resulted in limited restoration of oxidative and inflammatory biomarkers. Taken together, the findings suggest that the gastroprotective effects of these quinoline derivatives arise from a combination of antioxidant and anti-inflammatory actions, with compound **4** showing the most comprehensive profile of activity. Nonetheless, the overall magnitude of several biochemical changes was modest, and the biological relevance of these differences should be interpreted with caution. Therefore, while compound **4** emerges as the most promising candidate among the tested derivatives, its therapeutic potential should be considered preliminary and warrants further pharmacokinetic and toxicological evaluation.

## Data Availability

All raw experimental data supporting the findings of this study will be deposited in the public repository Harvard Dataverse (https://dataverse.harvard.edu/, acessed on 3 August 2026).

## Supplementary Materials

The following supporting information can be downloaded at https://www.mdpi.com/article/10.3390/molecules31162815/s1: Scheme S1. Synthesis of 2-phenylquinoline compound; Scheme S2. Synthesis of quinolines structurally related to 2-PQ: 2,4-diphenylquinoline (1), 2-(4-methoxyphenyl)quinoline (2), 2-phenylquinoline-4-ol (3) and 4-methoxy-2-phenylquinoline (4).

## Abbreviations

The following abbreviations are used in this manuscript:

2-PQ	2-Phenylquinoline
ANOVA	Analysis of variance
CEUA	Comitê de Ética no Uso de Animais
CONCEA	Conselho Nacional de Controle de Experimentação Animal
DMF	Dimethylformamide
DTNB	5,5′-Dithiobis-2-nitrobenzoic acid
ELISA	Enzyme-linked immunosorbent assay
Et_3_N	Triethylamine
FOX	Ferrous oxidation–xylenol orange
GSH	Reduced glutathione
H_2_RAs	Histamine H_2_ receptor antagonists
HCl	Hydrochloric acid
i.p.	Intraperitoneal
LOOH	Lipid hydroperoxide
MeI	Iodomethane
NMR	Nuclear magnetic resonance
NSAIDs	Nonsteroidal anti-inflammatory drugs
p.o.	Per os (oral administration)
PPIs	Proton pump inhibitors
PUD	Peptic ulcer disease
ROS	Reactive oxygen species
SEM	Standard error of the mean
t-BuOH	Tert-butanol
t-BuOK	Potassium tert-butoxide
TFA	Trifluoroacetic acid
TLC	Thin-layer chromatography
TNF-α	Tumor necrosis factor alpha
