# Supplementary material for: Bioinspired Quinoline-2 Derivatives Based on the Natural Alkaloid 2-Phenylquinoline from Conchocarpus longifolius (A.St.-Hil.) Kallunki & Pirani: Pharmacological Evaluation of Their Gastroprotective Potential
Source: Molecules. 2026 Aug 13;31(16):2815. doi: 10.3390/molecules31162815 (PMC13515230; doi:10.3390/molecules31162815)
Supplement: Supplementary file 1 [file molecules-31-02815-s001.zip › molecules-4447656-supplementary.pdf]

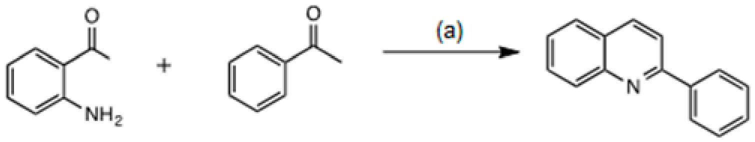

Scheme S1. Synthesis of 2-phenylquinoline compound.

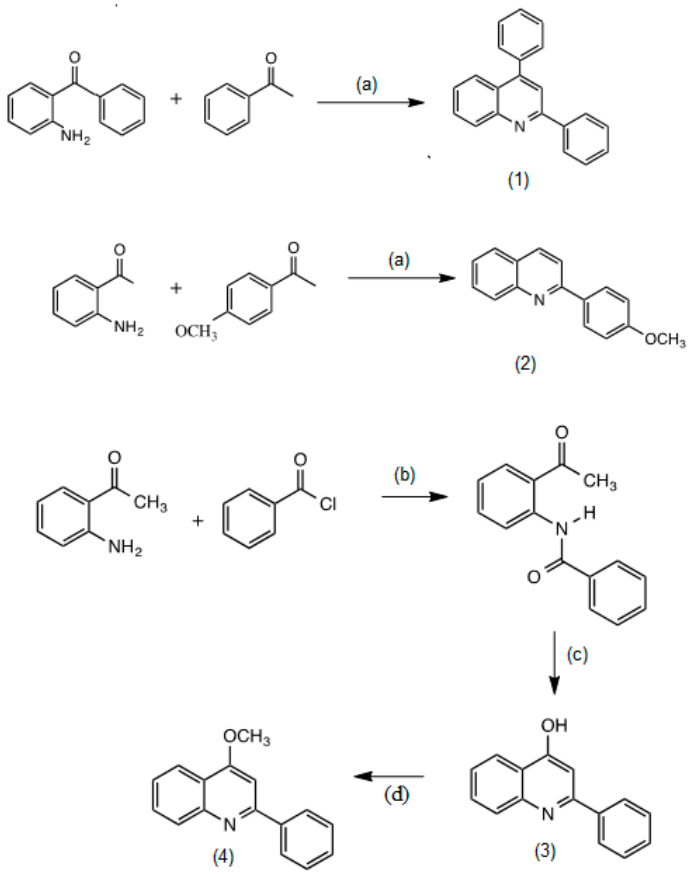

Scheme S2. Synthesis of quinolines structurally related to 2-PQ: 2,4-diphenylquinoline (1), 2-(4-methoxyphenyl)quinoline (2), 2-phenylquinoline-4-ol (3) and 4-methoxy-2-phenylquinoline (4).
